# How to Manage Red Alert in Emergency and Disaster Unit in the Hospital? Evidence From London

**DOI:** 10.3389/fpubh.2021.634417

**Published:** 2021-09-21

**Authors:** Mohammad Heydari, Kin Keung Lai, Zhou Xiaohu

**Affiliations:** ^1^Business College, Southwest University, Chongqing, China; ^2^International Business School, Shaanxi Normal University, Xi'an, China; ^3^The School of Economics and Management, Nanjing University of Science and Technology, Nanjing, China

**Keywords:** consultation planning, patient-flow, Imperial College Healthcare NHS trust, SCM, entropic estimator

## Abstract

This research gave an overview of coordinated hospital planning issues. In these issues, patients desire an arrangement for different source types, ideally as quickly as time permits. This field of context has just picked up academic interest, despite its reality since 1995. The way may discover a clarification for the above aspect that managing the hospital sources is regularly performed separately without taking a bigger picture. Therefore, it is particularly valid if the sources are situated in different departments. Another subsequent clarification may be related to the notoriety of the patient flow context. Hence, patients shouldn't be planned in these issues to be queued for another source or leave the system in case of their satisfaction of solicitation for the services at a particular source. The primary contribution of the present research is assisting present and new scholars via enumeration for every progression of the study of accessible decisions in the present context. Such means could be represented by major references for scientists to discover such studies endeavors tailored to their respective requirements. This principle removes the message: scientists ought to consistently coordinate their decisions concerning the setting, the capacity, and the approaches, as not all blends are conceivable.

## Introduction

Emergency logistics research includes the emergency allocation, transportation, and distribution of materials for emergency relief in disaster areas after the occurrence of sudden natural disasters and social hazards such as earthquakes, floods, hurricanes, mudslides, outbreaks, major traffic accidents, production accidents, and terrorist attacks and the delivery of personnel.

Compared with conventional commercial logistics activities, the cost and time of emergency logistics are contrary to each other, and time is more dominant in the goal. Therefore, it has the characteristics of sudden and unpredictable, stochastic demand, the urgency of time constraints, peak value, a weak economy, unconventionality, government, and market participation. In this case, the particularity and continuity of different disaster relief stages should be taken into account in the study of associated issues and the construction of models.

Prompt admission to health care has been considered to be vital for satisfaction and protection. Ever-increasing humans are employing the health services for planned and unplanned care; therefore, roust techniques would be required for addressing excessive demands (1). In situations where humans are not migrating via the system properly, it might also imply that other people experience delays and inaccessible care, resulting in considerable damage (2, 3). Such a condition can increase healthcare charges through the inability to utilize the expert staff time and grow the duration that humans are employing the services (4).

Hence, it will be necessary to ensure the existence of a match between capacity and demands for keeping the patients' safety, dignity, as well as privacy that may be conducted via ensuring on-time availability of the senior personnel, accessible diagnostics and examinations, true team-work, sufficient supports from inpatient specialties, and easily reachable hospital beds as an instance (5).

This research provides an overview of the context of combined hospital planning problems. The patients should go to several sources in a hospital to enjoy the complete therapeutic options for these problems. Hence, every patient will be allocated a particular path in a sub-set of sources, and thus every step of the path should be planned. The essential purpose of the recognized issues has been to enjoy every patient entirely from each stage of their paths during the intended due date. In this way, each patient will be given on-time care. Such conditions are essential because a delayed therapeutic or diagnosis might also cause reverse health consequences. In addition, in the case of the combined planning, hospitals possibly enhance the patients' satisfaction by developing an even patient flow to visit a couple of hospital departments. However, for structuring the developing context in this area, experts in the field suggested a classification scheme. They employed it to classify each scientific project on combined hospital planning reported from 1995 to 2016. Thus, the outcomes have been fascinating, though pathway principles like the diagnosis-associated groups or medical pathways were nearly presented many years ago. According to the classification scheme, most related studies have been conducted recently. Therefore, combined hospital planning is presently practicing in the scientific context. Finally, they appear to have recognized that removing the information silos in the hospitals is not optional; however, an absolute must tends to maximize the overall performance.

This research described an estimation process that relies on a 2-stage schema. It has been appropriately customized for the problems regarding the patient-flow dynamics and SCM (6). The merit of this new estimation technique is providing a way for the automatic fabrication of the generic estimation algorithms (7).

This research addressed formulating and solving the following estimation and forecasting problems. A specific patient-flow algorithm of the hospital with a collection of nominal parameters and specific sometimes-stamped sensory data with the uncertainty stage described by the entropy fluctuation tensor (8), a state estimate/forecast of the flow would be generated that is demanded by planning. Then, this estimate/forecast would be established by a recursive 2-stage schema wherein the estimates have been regarded as a perturbation of parameters. The advantage of the proposed estimation method is that it automatically constructs the generic estimation models. Fisher's information transformation would confirm the association between this forecasting schema and the standard filtering algorithms.

## Literature Review

From the article of Odegaard F, Chen L, Quee R, and Puterman M.L (9), the same issue can be seen with the hospital we investigated. Vancouver General Hospital (VGH) was facing a shortage of porters with a centralized dispatch system handling 300,000 cases per year. However, they have allocated several decentralized porters to handle exceptional cases. Instead of using a queuing algorithm like the research via Dershin and Schaik (10), which analyzed the impact of the porter services on the patients' waiting time, the research team adopted the simulation approach to deal with the problem. They attributed the inefficiency of the patient-flow problem to the unavailability of the porter. It then caused a delay in handing over patients to the CT room and their transportation from one ward to another. In our terminology, we will say the searching time of the porter flow is long for that hospital. The research also did qualitative research on the actual porter performance as they perceive the data that they collected are not highly accurate. They named this straight observation of porters and dispatch systems “*shadowing*.” They believe that the data cannot reflect some critical time like pickup time and clear call time and when the bottleneck occurs.

This research also provided insights into the challenges in porter operations, including communication problems, inefficient allocation of porter routines and supporting software, and lack of performance metrics and measurements.

In the second half of their research (data analysis and simulation), the researcher pointed out the mismatch between demand and capacity. They found that a significant difference concerning porter capacity occurred on Thursday and Sunday. The unused porter capacity was then lost as he or she couldn't be carried to the following hour. Better planning and optimisation of the workforce were craved in the research. The prolonged dispatch times were due to inefficient calculation in the dispatch system, hardware and software constraints, and the porters' unavailability. The prolonged pickup times result from the patients not being prepared when the porter arrives, porters arriving without the critical instruments, or their want for further porters for completing the transfer. A reason for pickup prolongs could be stemmed from the absence of communications between the diagnostic wards, dispatchers, and areas.

In the simulation part, the researcher made two critical changes in the current situation: (1) decentralization and (2) optimisation of staff plans. In the first set, a portion of porters will stay in higher demand in specific departments. In the second set, an optimal daily porter shift plan was employed. The improvements were made without additional costs^1^ by reassigning porters to shifts. Moreover, they could decide where to add the new porter. Tables 1, 2 have shown a comparison between our hospital and the VGH.

**Table 1:** Illustration of the similarities of NHS Trust and VGH.

**Similarity**	**NHS trust**	**VGH**
The number of cases per year	32,000	30,000
The number of available porters	60–80	50
Inpatient - Priority	3 categories: normal, urgent, very urgent	3 categories: STAT, ASAP, routine
Are porters involved in all medical services?	No	No
Clear-call time	Short	Short

*Source: Authors*.

**Table 2:** Illustration of the differences between NHS trust and VGH.

**Difference**	**NHS trust**	**VGH**
Our nurses and doctors are involved in porter services?	No	Yes
The mode of the porter dispatch system	Centralized	Decentralized
Communication tools	Walk-talkie	Pager
Geographical structures	High buildings in a small area	A large area
Facilities	Number of departments	

*Source: Authors*.

According to the document from Queen Mary Hospital (11), the hospital was facing long porter service time. They attributed the problem to different areas. They acknowledged that the geographical constraint was the cause of delayed provision of services like elevators' capacity and inefficient hospital structure. They pointed out the peak hours are 1–2 p.m. and 5–6 p.m. The possible solutions were to deal with the peak hours, with better communication and team-work between designated decentralized porters, centralized porters, and security teams with the coordination of the control center.

Khorram-Manesh A, et al. in their study, showed the requirements for a reliable plan based on risk and vulnerability analyses: knowledge of routes, spaces, etc., collaboration with other agencies to optimize source availability (12), plan for vulnerable groups, role identification within each organization and between agencies, realistic training/exercises, evacuation of patients may contain the caretaking of relatives, essential to note safety and security issues, communication through regular briefing and functional communication system, knowledge of triage in the evacuation, staff continuity and adequate supplies, effective leadership and central command, surge capacity measures and early decision-making, use of volunteers, and emergency departments should plan for continuous patient arrival during evacuation—shelter-in-place results in critical and prolonged periods of shortage.

As Khorram-Manesh A, et al. stated, a disaster plan shall be based on risk and vulnerability analyses (13): action cards for staff, training, reliable internal and external communication, information sharing and delivering, electrical power source, supplies delivery, collaboration via other agencies, follow-up of the psychological trauma, positive reinforcement with hand-written journals and escorting prehospital teams with drug supplies, and the role of private facilities.

Khorram-Manesh A, et al. in their study, showed the following: information delivery and information sharing (14), proximity to other hospitals, reliable communication, complete backup system, access to field hospitals, access to electrical power sources, collaboration with other agencies, including armed forces, ICS with stable leadership and decision-makers, internal supporting systems in hospitals for water, heat, and food, coordination and collaboration between staff, and patients require assistance and need training on evacuation routines.

As we deal with 600,000 records of a dataset, we have to employ data handling techniques such as data warehousing and *OLAP*^2^ strategies. Vasilakis (15) provides insights into the automation of the algorithms and procedures to estimate a wide range of parameters for the simulation. The computational complexity of analyzes and interpretation can be reduced.

Examples include the Health Foundation's learning report, the respective case studies describing the work from 2 NHS trusts for addressing the poor patient flow in unplanned care (16, 17). *It is notable that “Patient-flow-associated approaches”* represent all techniques searching to recognize obstructions in services and the way of their effective identification^3^.

On the one hand, governments are trying to reduce their health care costs and increase healthcare systems' efficiency. The present article addressed a major hospital process in the UK wherein the inpatients present, patient-flow management. Patient-flow management influences the entire operating system of the hospitals as inpatients generally allocate several sources to them that include facilities and spaces such as elevators. Therefore, a main hospital in the UK has been investigated on the patient-flow management issues and compared with a hospital in Canada in terms of benchmarks. Moreover, the differences and similarities of both hospitals have been recognized, and then congestion of the patient flow has been examined in two main steps. First, operation data from the hospital has been checked, and bottlenecks and the most famous paths have been specified. In addition, qualitative comments of the operations from the hospital have been obtained for consolidating data analysis. Furthermore, we utilized the clustering methods. Finally, relying on the outputs of algorithms for improvement, several probable recommendations have been illustrated.

The Health Foundation was fascinated by the further perception of how approaches to address patient flow might assist in developing results in unplanned (emergency) cases. Such a condition due to the existence of >100 million NHS visits or calls every year associated with unplanned care, accounting for 1/3 of each NHS endeavor that includes above half of the charges (18, 19). Sometimes, the issue of matching supply and demand (red alert)^4^ for OR time is viewed from the lens of time scales. In this paradigm, decisions such as the types of surgical procedures performed, the number of ORs, and how each OR is equipped are long-term decisions (typically made once every few years).

For instance, NHS England's latest overview of unplanned care (20) discovered that nearly 24,000,000 calls had been reported to the NHS emergency care phone services (21, 22), 7,000,000 emergency ambulance journeys (23), 22 million visits to emergency and accident (A& amp; n E) minor injury units, urgent care centers as well as departments (24), and 5,000,000 emergency admissions to the hospitals in England (24). These figures demonstrate various emergency services accessible and the scope of their uses (25).

Nonetheless, the analysis of a study of the usage of patient-flow-associated approaches for improving emergency care services results discovered that many publications of the emergency care centered on particular departments instead of demonstrating broader systems, pathways, and organizations (26). Hence, strategies to analyze and modify the patient flow might also be greatly utilized across more than one branch or department.

Consequently, to supply exact extra instances of inspecting and developing flow, the Health Foundation made decisions for compiling studies using the above procedures in the organizations or ways of care instead of concentrating on unplanned care. Therefore, it is possible to use learning for emergency services and several aspects of health care.

The present research introduces an estimation process to rely on a 2-step graph. This process is appropriately customized for issues regarding the patient-flow dynamics as well as SCM.

The obtained documents can address the following question:

*What empirical contexts exist regarding the techniques to analyze or modify the patients' movement through organizations or paths of care?*

Given the small area but densely built buildings in the hospital, congestion always happens in peak hours which causes the total delivery time to be much longer than expected. We have communicated with the hospital, and they mentioned the process of the porter services and several problems they encountered during their operations.

## Strategy and Planning Procedure

In this phase, researchers know most restrictions and boundaries of the selected context. Hence, we enlarge the algorithm from a condition of restrictions to an entire optimisation algorithm with an objective function. Put differently, this part poses the scientist's question of what desires to be optimized and the way of performance definition in hospital. However, in the combined health care, experts in the field significantly emphasized developing the patient-oriented operations in the hospitals for augmenting the patients' satisfaction stage. Hence, when planning a collection of requests for consultations, the patient probably wishes a plan which minimizes the time-span between the first and last consultation regarding the medically necessary time for recovering from a process. In addition, being assisted in a hospital quickly has been considered one of the enormous determinants of the patients' satisfaction (27). Table 3 reports that each study in the NHS Trust publications is devoted to minimizing the path completion time of maximizing the patients' satisfaction.

**Table 3:** Taxonomy relies on the objective function.

**Objective function**		**References**
Different types of the	One objective	(28–34)
objective function	Multiple objectives, with weights	
	Multiple objectives, with different stages	
Goal	Minimize access time	(28, 32, 35–37)
	Minimize idle time of sources	
	Maximize satisfaction	
	Minimize time to finish all assignments (or minimize waiting time among two consecutive steps)	
	Maximize the number of patients planned (scored and unscored patient priority)	
	Other objective function	

Moreover, since hospitals want to be more affordable and experience budget cuts (38), profit maximization has been considered a hot topic. Therefore, the aim of the NHS Trust may extensively be categorized into two groups. In other words, hospitals may select the following the objectives of the combined healthcare publications and intensify the patients' satisfaction, minimize access time, or minimize the completion time of each task. Several hospitals maximize the profits by enhancing the number of patients planned, increasing the contribution margin, or minimizing the idle time of sources. The two kinds of objective function may be legitimate concerning the setting, and NHS Trust has approved efficacious. Therefore, the latter additionally justify the articles with numerous aims through allocating the weights to all parts of the objective function or through numerous optimisation phases. Consequently, scientists can allow the hospitals to make decisions of which objective function kind is intended. One of the instances may be discovered by Bikker et al.'s (28) study wherein the hospital may select the allotted weights to minimize the access time and minimize the idle time of the physicians supplying the consultation.

In the earlier decision phases, each objective function and each constraint of the issues will be described step by step. Therefore, options concerning the decision stage, the context, patient mix, and scope described the issues that the investigator wishes to investigate, and the optimisation algorithm is presently perfect. Scientists select which procedure is fantastic to obtain the intended objective. In doing this condition, scientists need to select a planning technique or planning approach. The latter represents this research to the differentiation of the online with the offline planning. When a request is received for collecting consultations, planners have two options considering their response times to the request. Put differently: they may directly respond with a time and date for the requested consultations. Such a condition refers to the fact that planning will be a sequential method wherein the patients receive consultations at the arrival time of their requests. Planners may additionally desire to wait and gather requests for consultations on the waiting list. Then an algorithm would be utilized to choose patients from the mentioned list. In inconsistency with the consultation contexts (39), the earlier planning method will be mentioned as online planning, and the latter will be mentioned as offline planning. The above classification shouldn't be stressed with the distinction between offline and online operational decision ranges mentioned. The offline decision stage would be described as the planning before a workday. Finally, the online decision stage represents when consultations need to be planned or re-planned in the course of the workday. Selecting the planning method has not been considered a lightweight assignment because each implies a unique hospital algorithm.

Table 4 indicates that most chosen articles employ an offline planning approach for serving the patients that may be defined through a reputation of techniques, which could not be utilized in the online trend due to the restrictions considered for the computation time. In case of the application of an exact approach like a branch and bound, re-evaluating the algorithm every time a novel request for a consultation is obtained would not be possible (31). In fact, in applying the waiting lists, planners must note the patients cannot remain on a list for a prolonged duration because the patients' satisfaction would decline by enhancing the patients' urgency stage (42). Furthermore, under outpatient conditions, another risk is posed: patients would visit the emergency department for quicker treatment (43).

**Table 4:** Taxonomy rely on the planning methods.

**Different Planning**	**References**
Online system	(28–30, 40, 41)
Offline planning	

While selecting a planning procedure, exceptional alternatives would be reachable on the list. Several such selections present optimum solutions (the exact approaches), whereas others solely present the near-optimal solutions, that is, heuristics. Therefore, it is generally tried to discover optimized stability in a trade-off of the solution quality with the computation time (31). When we face an online planning approach, this technique should be employed every time a current request is received for a consultation. Hence, computation time must be short of restricting the attempts to solve real-life examples to ordinarily (meta)heuristics.

Nonetheless, the mentioned techniques search for a small phase of the search space that hardly leads to an optimal solution. On the contrary, hospitals intend to supply a therapeutic plan, enhance patient satisfaction, and keep inappropriate throughput stages. Moreover, in offline planning, numbers of the patient, for which consultation must be planned, is greater, and consequently, the complicatedness of the problem enhances quickly. In addition, the best optimal solution frequently would remain just something to want to. Table 5 reports the argument outcome. It indicates that the variety of accurate planning strategies is relatively restricted compared to the number of articles investigating a near-optimal solution. The set of the near-optimal solution strategies is similarly dominated via the well-known metaheuristics, like the genetic algorithms and tabu search and multi-agent approaches. Meta-heuristics search a solution vicinity (or a set of solutions) for creating more acceptable solutions. The multiagent techniques assign one of the agents to each stakeholder in the planning procedure with specific necessities and interests. Here, the aim has been regarded as creating a plan compatible with the priorities and restrictions of each agent (49). Therefore, the plan generation may result from various methods like different techniques utilization of the optimistic heuristics. A popular method in the procedure has been proposed to simulate a combinatorial auction wherein the auctioned items would be the time-slots supplied through the source agents. Hence, willingness to pay for every item or an integration of the items would impress an optimisation goal. To fully assess the articles using the multi-agent theory in the healthcare sector up to 2008, Isern, Sanchez, and Moreno can be mentioned (50).

**Table 5:** Taxonomy relies on the approach.

**Approach**		**References**
Heuristics	Metaheuristics	(29, 30, 44–47)
	Other heuristics	
	Planning rule (e.g., FCFS^a^, FCRS^b^)	
	Multi-agent theory (by auction)	
	Multi-agent theory (by another method)	
Exact algorithms	IP^c^/LP^d^/MILP^e^	(48)

a *First come, first served (FCFS)*.

b *First come-random-serve (FCRS)*.

c *Integer linear program (IP)*.

d *Linear program (LP)*.

e *Mixed-Integer Linear Programming (MILP)*.

Moreover, chosen references in the present research are the extension of a study in (50). In addition, the multi-agent strategies additionally demonstrated that source coordination might be hindered because every source can be allocated a sourcing agent. Such a condition would be contrary to the precise approaches that demand coordinated decision-making. Consequently, scientists must be very cautious of the procedure's implication as every procedure is straight coruscated with the level of integration into the hospital. However, the latter particularly holds real in envisioning implementing the developed approach through the investigator by the hospital.

Notably, strategies like MDP^5^ and queuing theory have been not shown by Table 5, which may be defined by the fact that each strategy as a substitute relies on the patient-flow rather than planning methods. According to the queuing theory, patients will go straight to the subsequent source. Thus, it aims to computation the common waiting times for the patients to undergo the system [(51, 52), as an instance]. A Markov decision process is crucially a sequential decision algorithm, which illustrates a system being in a state. Moreover, because of an action, the system transfers into the next state Such a situation takes place based on a transition function, illustrating the probability that an action in the stateleads to the state (53). In this regard, Garg et al. (54) algorithm, as an instance, the patient transition method via a health care system assumes that a patient strikes from one stage to the next stage without a need for consultation of the subsequent source. The same strategy may be discovered in Hulshof et al.'s (55) study wherein the patients flow to the subsequent source and thus queue or finally leave the system. Schaefer et al.'s (56) study will be referred for additional instances.

Nonetheless, it is possible to employ the explanations of Markov decision processes to conclude that inpatient planning, every new consultation, may be described as an action leading to a novel state, which may be illustrated through a vector of the previously booked patients. Therefore, it is utilized for single-source planning [Gocgun et al. (57) for the latest instance in the computed tomography]. Nevertheless, there are few articles to apply a Markov decision process to plan patients on numerous sources. A rationalization for this gap in the conducted studies may be found in the computational complicatedness of techniques for solving a Markov decision process (53).

To assist patients to receive different medical services, porters are employed to migrate patients from one department to another around the hospital. Besides, porters have to perform other tasks such as

- Managing sickbeds.
- Delivering materials such as sickbeds, life support machine, oxygen equipment, medical products.
- Other miscellaneous stuff like cleaning, changing sickbeds.

**Figure 1:**
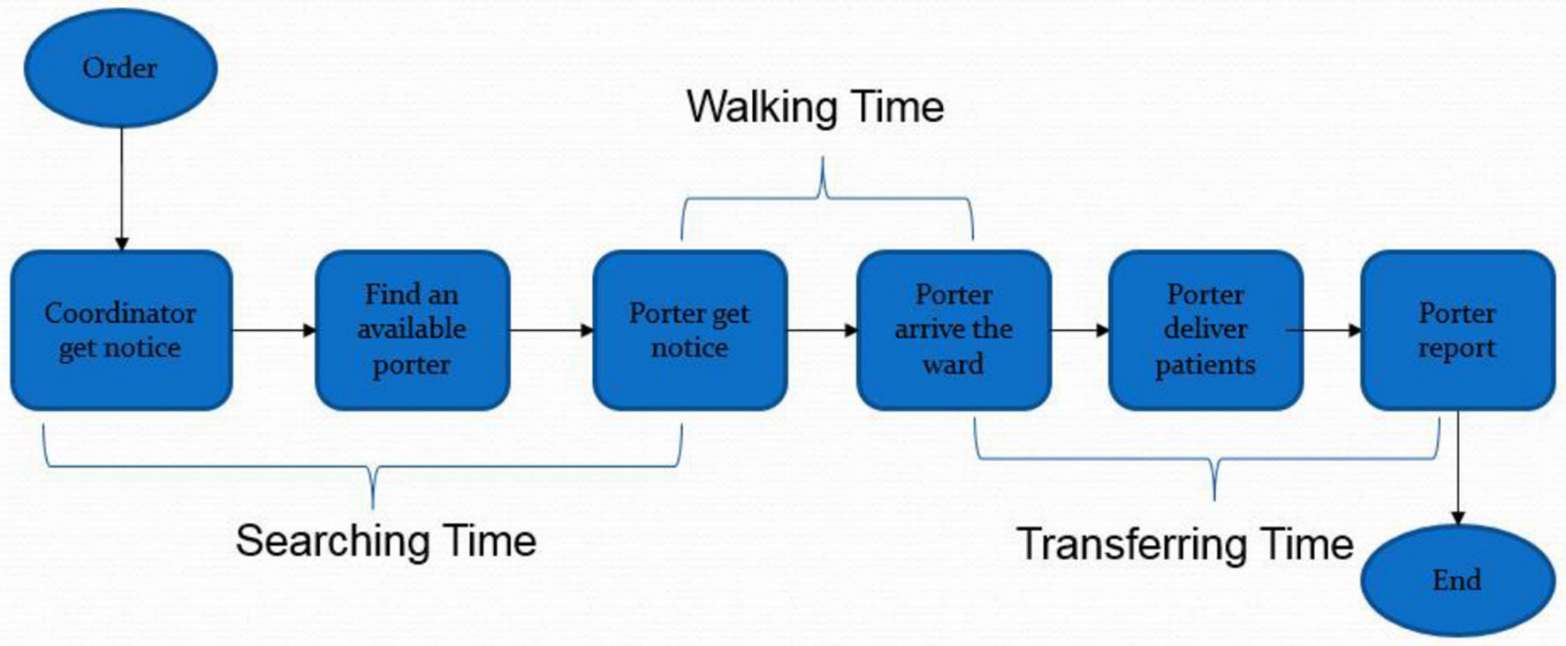
Process of patient-flow. Source: Friesen et al. (58).

Porters work with orders to perform the above tasks, as shown in Figure 1.

When a patient needs to be migrated, or some material is required somewhere, an order will be placed in a centralized system with the notification of coordinators by nurses or porters. The urgency, requirements, and other information will be entered into the computer. When an order is received, the system will dispatch the nearest porter to the ward to perform the task. If more cases are present, the priority of the cases will be relying on the urgency and the waiting time of the case. On some occasions, the coordinator will assign the porters themselves without considering the system's assignment. They will pick up the notice and find an available or idle porter by walker talkie. The time needed to find an available porter is called “*searching time”* for porters (mean = 5.37, standard deviation = 10.66). After receiving a task, the available porter will be occupied, and he or she will walk to the starting ward to pick up the patients; the “walking time” is then recorded (mean = 4.00, standard deviation = 3.56). The porter will subsequently deliver the patient to the destination and report to the coordinator. The time between starting to move to the patient and reporting the finished order is called “*migrating time”* (mean = 9.025, standard deviation = 7.13). Usually, the time of reporting is short, so that it could be negligible.

## Methodology

This article illustrated a 2-stage schema to approximate the process rate variables appearing in a general supply chain dynamic algorithm. Therefore, the 1st stage estimated the parameter “dependency functions” ***ψ***_***j***_**(*****t, k*****)**. Applied as the inputs for the 2nd stage, wherein a perturbation of parameters **Δ*****k*** would be estimated. Then, an algorithm has been provided for computing the estimates ***ψ***_***j***_**(*****t, k*****)** for situations where the system state observations are carried out at the discrete instances of time. Ultimately, its correlation to the Fisher information matrix has been shown.

This research addressed formulating and solving the following estimation and forecasting problems. A specific patient-flow algorithm of the hospital with a collection of nominal parameters and specific sometimes-stamped sensory data with the uncertainty stage described by the entropy fluctuation tensor (8), a state estimate/forecast of the flow would be generated that is demanded by planning. Then, this estimate/forecast would be established by a recursive 2-stage schema wherein the estimates have been regarded as a perturbation of parameters.

Fisher's information transformation would confirm the association between this forecasting schema and the standard filtering algorithms.

### Entropic Estimator in a General SC as an Algorithm^6^

We algorithm an SC as one of the membrane networks operating on the information and goods flow (7). It is an operation dynamic of an element of an SC that has been encoded as the rules like:^6^

(1) $$\begin{array}{r} a_{1}^{j}\xi_{1}⊗a_{2}^{j}\xi_{2}⊗\ldots ⊗a_{N}^{j}\xi_{N}\overset{k_{j}}{\to }b_{1}^{j}\xi_{1}⊗b_{2}^{j}\xi_{2}⊗\ldots ⊗b_{N}^{j}\xi_{N},\\ j=1,\ldots ,r, \end{array}$$

where ξ_1_, …, ξ_*N*_ refer to the money, goods, and services labels that flow across the element, $a_{i}^{j},b_{1}^{j},i=1,\ldots ,N,j=1,\ldots ,r$ stand for the input and output stoichiometric coefficients that indicate the numbers of units of every item entering *j* − *th*step of the process. Moreover, the flow rate constants of *k*_*j*_, *j* = 1, …, *r*, stand for the parameters characterizing the underlined process's storage capacity and flow constraints (For more information see Appendix A).

The process illustrated in (7) generates a dynamic algorithm that is presented here:

(2) $$\begin{array}{l} \frac{d}{dt}\eta \left(t,k\right)=\bar{A}\left(k\right)\eta \left(t,k\right)+\bar{B}\left(k\right)\bar{u}\left(t\right). \end{array}$$

Here matrices **A** and **B** are dependent on the rate coefficients, *k* = (*k*_1_, …, *k*_*r*_). It is assumed that no changes will be made in rate coefficients over time; that is,

(3) $$\begin{array}{l} k=0. \end{array}$$

It has been found that the rate coefficients{*k*_*j*_}won't be precisely known, and thus, they can be defined by experimentations. However, this paper tends to set up a schema to estimate the above algorithm parameters. Therefore, (2) would be firstly differentiated based on *k*_*j*_, and consequently, the differentiation order would be interchanged (considering adequate continuity conditions) for getting:

(4) $$\begin{array}{r} \frac{d}{dt}\psi_{j}\left(t,k\right)=\bar{A}\left(k\right){|}_{\bar{k}}\psi_{j}\left(t,k\right)+\frac{\partial \bar{A}\left(k\right)}{\partial k_{j}}{|}_{\bar{k}}\eta \left(t,k\right)\\ +\frac{\partial \bar{B}\left(k\right)}{\partial k_{j}}{|}_{\bar{k}}\bar{u}\left(t\right)+Gw\left(t\right), \end{array}$$

Where $\psi_{j}\left(t,k\right):=\frac{\partial \eta \left(t,k\right)}{\partial k_{j}},\bar{k}$ represents the mean value of the parameters *k* and a “*noise term' Gw*(*t*) would be added for investigating this issue that there is not information of the exact rate coefficients $\bar{k}$. As seen, *w*(*t*) represents a martingale process with zero-mean and co-variance equal to the inverse of the information matrix **Λ**, which measures knowledge uncertainty on the above rate coefficients.

### Optimal Forecast Engine

**Figure 2:**
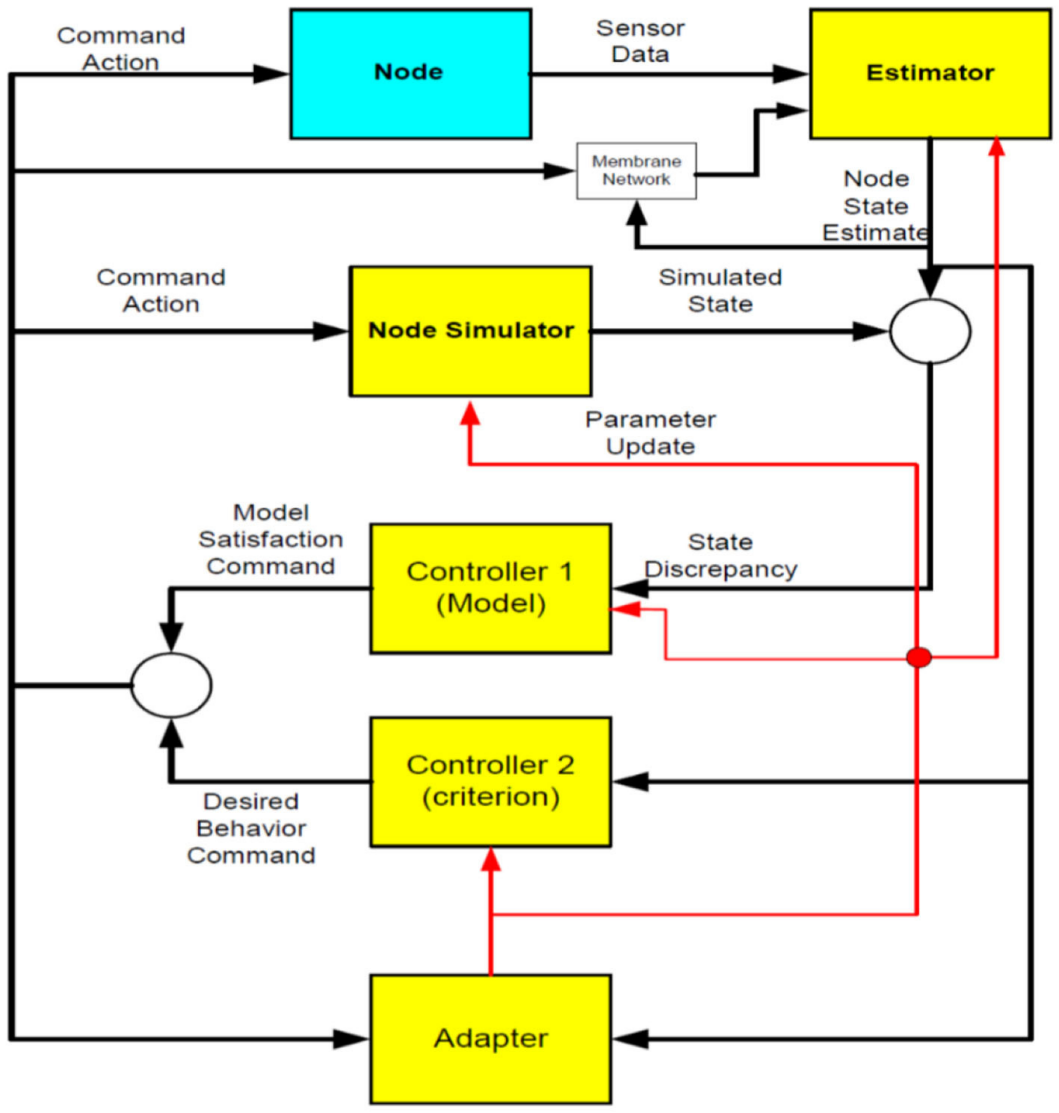
Block Diagram of a control cluster. Source: Authors.

Figure 2 displays the operation process of a control cluster. Firstly, the Sensory data from the controlled system (Node) and the Network would be fed to Estimator. Then, Estimator would output a state estimate, and the adapter applies the obtained estimate of a system state for computing an estimate of the algorithm parameters. The obtained estimates would be employed to update the algorithm. Node simulator generates the other state with the use of an algorithm with the currently available parameters. There are differences between the novel estimate of a system and simulated state; the state will be input into Controller 1. Then, a function of Controller 1 would be computation of a control law, which compensates for algorithm discrepancy. Moreover, Controller 2 employs the estimate of a system state for computing an optimum control law, which minimizes a specific criterion.

It is assumed that it is possible to compute a total state estimate of the system $\hat{\eta }$. Therefore, according to Eq. (4), we demonstrate a system dynamic via the stochastic differential equation:

(5) $$\begin{array}{l} dx\left(t\right)=Ax\left(t\right)dt+Bu\left(t\right)+Gdw\left(t\right), \end{array}$$

Where $x\left(t\right):=\left[\begin{array}{c} \psi_{j}\left(t,k\right)\\ \hat{\eta } \end{array}\right],A:=\left[\begin{array}{c} \bar{A}\left(k\right)|\bar{k}\\ \frac{\partial \bar{A}\left(k\right)}{\partial k_{j}}|\bar{k} \end{array}\right],and\;B:=\left[\frac{\partial \bar{B}\left(k\right)}{\partial k_{j}}|\bar{k}\right].$

Hence, an observation algorithm^7^ consistent with SC utilization will be presented as

(6) $$\begin{array}{l} z\left(t_{n}\right)=Hx\left(t_{n}\right)+v\left(t_{n}\right), \end{array}$$

and here, measurements have been made at discrete time instances *t*_*n*_, *n* = 1, 2, …, not inevitably with the equal space and *v*(*t*_*n*_) are a zero-mean discrete martingale procedure with co-variance *R* independent of *w*(*t*) that algorithm uncertainty stage during the demand observations.

#### Time-Update Between Measurements

Co-variance matrix ∑(τ|*t*_*n*_), and conditional mean $\hat{x}\left(\tau |t_{n}\right)$ where *t*_*n*_ ≤ τ ≺ *t*_*n*+1_, are propagated according to the following equations:

(7) $$\begin{array}{l} \hat{x}\left(\tau |t_{n}\right)=F\hat{x}\left(\tau |t_{n}\right)+Bu\left(\tau \right) \end{array}$$

(8) $$\begin{array}{l} \sum \left(\tau |t_{n}\right)=F\left(\tau |t_{n}\right)+\sum \left(\tau |t_{n}\right)F^{T}+GQG^{T} \end{array}$$

#### Measurement Update

It should be mentioned that discontinuity will be observed in the trajectory $\hat{x}$ simultaneously *t*_*n*_.; therefore, Eqs. (9) and (10) show computation of the covariance ∑(*t*_*n*+1_|*t*_*n*+1_) as well as conditional mean $\hat{x}\left(t_{n+1}|t_{n+1}\right)$ following observation at the time *t*_*n*+1_:

(9) $$\begin{array}{l} \hat{x}\left(t_{n+1}|t_{n+1}\right)=\hat{x}\left(t_{n+1}|t_{n}\right)+K_{D}\left(z\left(t_{n}\right)\right)-H\hat{x}\left(t_{n}|t_{n}\right)) \end{array}$$

(10) $$\begin{array}{l} \sum \left(t_{n+1}|t_{n+1}\right)=\left[I-K_{D}H\right]\sum \left(t_{n+1}|t_{n}\right) \end{array}$$

So that (discrete) Kalman filter *K*_*D*_ will be:

(11) $$\begin{array}{l} K_{D}=\sum \left(t_{n+1}|t_{n}\right)H^{T}{\left[H\sum \left(t_{n+1}|t_{n}\right)H^{T}+R\right]}^{-1} \end{array}$$

## Final Algorithm

### Association With the Fisher Information

For estimating k from η(t, k) as well as x(t), assume a perturbation in $\hat{\eta }$

(12) $$\begin{array}{l} \Delta \hat{\eta }\left(t,k\right)=\Psi^{T}\left(t\right)\Delta k+\hat{\eta }\left(t,k\right)\Delta t, \end{array}$$

Where matrix $\Psi^{T}\left(t\right):=\left[{\hat{\psi }}_{1}\left(t\right)\ldots {\hat{\psi }}_{r}\left(t\right)\right]$ with the entries achieved from $\hat{x}\left(t\right)$.

From (12),

(13) $$\begin{array}{l} y\left(t\right)=\Psi^{T}\left(t\right)\Delta k\left(t\right), \end{array}$$

**Figure 3:**
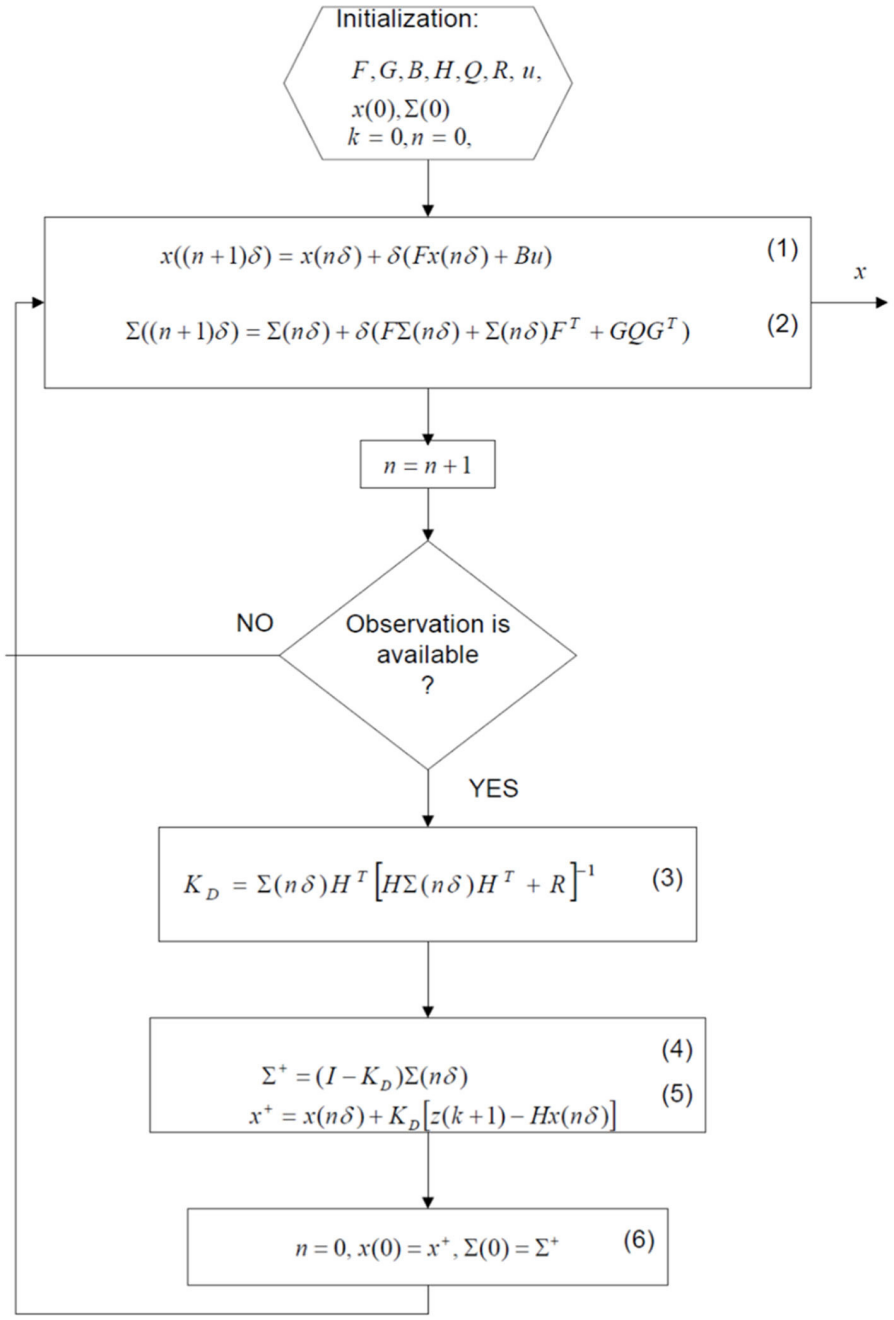
The algorithm flowchart.

Wherewith matrices and $\bar{B}$ assessed at the current estimate of *k* (see Figure 3).

Using continuous least-square estimation theory (59), the estimates equations, which minimize lost function, $V\left(k\right)=\overset{t}{\underset{0}{\int }}e^{-a\left(t-\tau \right)}\left(y\left(\tau \right)-\Psi^{T}\left(\tau \right)\Delta k\right)d\tau$ are as follows:

(14) $$\begin{array}{l} \frac{d\hat{\Delta k}}{dt}=P\left(t\right)\Psi \left(t\right)e\left(t\right)\\ e\left(t\right)=y\left(t\right)-\Psi^{T}\left(t\right)\hat{\Delta k}\\ \frac{dP\left(t\right)}{dt}=aP\left(t\right)-P\left(t\right)\Psi \left(t\right)\Psi^{T}\left(t\right)P\left(t\right), \end{array}$$

whereby explanation, $P^{-1}\left(t\right):=R\left(t\right):=\overset{t}{\underset{0}{\int }}e^{-a\left(t-\tau \right)}\Psi \left(\tau \right)\Psi^{T}\left(\tau \right)d\tau .$

As seen, matrix R(t), also known as the Fisher information matrix, will satisfy the following dynamics:

(15) $$\begin{array}{l} \frac{dR\left(t\right)}{dt}=-aR\left(t\right)+\Psi \left(t\right)\Psi^{T}\left(t\right). \end{array}$$

## Discussion

Overall, the hospital faces enormous congestion during the peak hours in the morning (10:00–12:00) and the afternoon (14:00–17:00). Several reasons can be attributed to the situation:

- Queuing for the elevators with limited capacity (broadly speaking, the more people use a lift, the number of times it stops, and the longer time it takes to reach a particular floor).
- Waiting for operational equipment and medical services.
- Lack of workforce.

The hospital handles 1,000 cases mentioned above every day, and around 80 porters are present in the hospital. In other words, each porter is responsible for four to five orders per hour, and each order takes around 15–20 min to finish optimally. (The hospital is targeted at meeting all the orders within 20 min). However, during peak time, 20% of the orders cannot be fulfilled.

From the hospital's perspective, most of the time, porters take much longer time than they are supposed to, especially during the peak hour in the morning (10:00–12:00) and the afternoon (14:00–17:00). This is mainly due to the long waiting time for elevators and operation equipment, thus lowering the efficiency of the hospital. The situation seems to be relieved slightly when it comes to the lunch hour. Meanwhile, some porters are described as idle during non-peak hours, which results in a waste of workforce.

Some problems with the hospital design are identified. First, sometimes porters with a patient have to travel almost 195 m to perform medical services and return. Second, in peak hours, the congestion is severe, so time is wasted on waiting. For instance, the efficiency of elevators will substantially decrease during peak hours due to a limited number of lifts and the number of times they stopped. Seemingly, it is hard to restructure the hospital immensely; instead, we can enhance the whole operation by re-planning the workforce and services. Through analyzing this project, we can realize whether we have to open up two diagnostic centers and whether we need to put DC in the middle of the hospital.

While deciphering the evidence scan outputs, numerous shortcomings should be investigated. For example, the scan is not comprehensive above 5,000 research posted about techniques to analyze or modify the patient flow throughout pathways, organizations, or the healthcare systems. This scan provides instances of the easily accessible posted empirical research for providing a flavor of the existing investigations for signposting the readers to fascinating materials and highlighting a number of the main implications for functionality to the unplanned care.

However, it is notable that the flow-associated approaches have been primarily demonstrated that are not qualified to be included in the evidence scan except that they rely on posted empirical studies. Therefore, multiple kinds of flow-associated techniques might be found, which won't be presented in this article due to the limited studies reported.

In the same way, alternate approaches have been solely covered in case of explicit search to analyze or modify the patient flow through the pathways or organizations (independent of the narrower unplanned care instances).

Another essential issue has been proposed by some scientists presenting elements regarding the actual procedures employed or step-by-step directions to replicate the patient-flow methods. Moreover, limited research has been reported on illustrating the influences of techniques to analyze or facilitate the flow on the consequences like patient satisfaction, safety, or costs. It won't imply that the obtained results would not be essential or influenced through flow-associated approaches, but there are limited documents on the posted subjects.

As for the problems with the extent of the documents, several shortcomings of the great studies have been presented. Many of the research covered in the evidence scan have been limited and navigated in specific hospitals, frequently outside the United Kingdom. Moreover, they usually employ quickly before and after designs without controlling other possible effects on the outputs. Additionally, some types of research have been published to evaluate different strategies for analyzing or altering the flow; hence, it would not be viable for concluding that a particular strategy has a higher or minor effect compared to the other.

According to the empirical documents, approaches to analyze and alter the patient flow enjoy potency in the healthcare system on a pathway-wide, organizational basis, and with the specific utility to the unplanned care. However, these approaches are heterogeneous, but researchers utilized them in some countries that have been accompanied by positive outputs, specifically enhancing throughput and reducing the duration of stay or waiting time.

The correlation of the NHS Trust concept to the combined health care publications has been illustrated. Combined health care or combined care represents several (frequently multidisciplinary) health care services for improving the continued care for each patient (60). Combined care seeks to create affordable, patient-oriented, and accessible care specifically for patients who suffer from complicated conditions (61). Therefore, scientists attempt to eliminate the information silos found between specialists or hospital departments (61). These attempts led to different approaches like focused factories and specialty clinics (62), using the physicians collaborating to define a necessary treatment for patients, and combined practice units, or one-stop shops (63). Hence, a combined hospital planning has been described as one of the extra dimensions in the scope of combined health care. Therefore, experts in the field utilized Drupsteen, van der Vaart, and van Donk's (64) framework and classified the NHS Trust concept as a functional integration approach. Publications of the combined health care particularly emphasized the efficiency of the combined care because each combined care approach seems to provide statistically more significant results (65). Such a condition translates to the NHS Trust, for which the scientists should approve good practical performance of their approach to the combined planning.

Since the NHS Trust deals with the path optimisation followed by the patients within a particular set of sources, it is necessary to differentiate NHS Trust concept and patient-flow publications. For the patient-flow studies, scientists frequently attempt to optimize the consumption way of a set of pre-defined sources (60). In this case, the patients don't need to consult on each visited source. Therefore, patients will go straight to the next source in satisfaction with their demands for services on the earlier source in these problems. Put differently, independent of the admission planning, planning does not occur. It aims to reduce the patients' waiting time, enhance the patients' efficiency, and stage the sources' capacity with demands for the desired service (37). Notably, it is not possible to see NHS Trust as entirely disjointed from the patient-flow problems. For instance, we may apply admission planning [for example, (20)] methods in an NHS Trust setting because of the planners' need to decide on the inpatients' admission time, even if each stage of the care procedure needs to be planned.

An NHS Trust has the same high stage as the flow-shop, open-shop, and job-shop planning problems regarding the above-said criteria. Actually, in a job-shop planning problem, the jobs should visit each machine and follow a pre-determined fixed sequence (63). Moreover, in an open-shop problem, the jobs' sequence to visit the machines will be inter-changeable, whereas, in a flow-shop problem, each job will follow a similar route through the shop. In case of restriction of the NHS Trust scope to just sequencing and planning patients on the hospital sources, it is possible to describe the NHS Trust as a flow-shop, open-shop, or job-shop planning problem precedence constraints. Azadeh et al. (29) and Vermeulen et al. (66) present some instances of the latter.

NHS Trust is one of the inherent parts of the consultation planning studies. Since the first attempts in this field date back to 1952, Bailey (67) and experts in the field quickly investigated it since then (50), and it is expected that the numbers of NHS Trust are entirely significant. Nevertheless, most articles regarding consultation planning addressed single-source planning in inpatient and outpatient planning issues (20). In this regard, Froehle and Magazine (45) and Van de Vrugt (39) referred to the scarcity of the studies transcending a simple clinic context. Hence, there are not enough documents on guiding planners in serving the patients who require visiting several care providers. Put differently, the new planning approaches ignored the complicated associations between the departments (68) on the tactical and operational stages (63). Thus, we hardly see combined management of the patient planning (46). In addition, planners have no understanding of the impacts of diverse integrations of the facility routings on the performance measures (38).

Consequently, the final plans can hardly be the best from a hospital perspective (45). On the other hand, health care planning may be observed from a source view (single-source planning) and a patient view (NHS Trust) (54, 61) so that the latter has been little examined. In several situations, the evolution toward the combined care is also knowingly stopped by departments tending to maintain the source calendars (66) locally.

An improvement in the patient flow may seem a relatively understandable thing, but departments or services frequently work in isolation. Some health systems and organizations have financed resources that pursue transporting the patients via the safest and most effective options; however, it is possibly the same thing necessary for influencing the flow. A more radical reorganization of the services and finance can be necessary because the patient pathway has been considered one of the major focuses instead of specific departments or services. Nonetheless, documents include relatively narrow interventions or a small scale, which usually do not cut across the sectors or services.

## Suggestions and Recommendations

The review suggested that five major principles of

- Whole system development,
- Diagnostic needs assessment and accurate time information,
- Adaptation of the range of approaches to the local contexts,
- Accounting for practicality, and
- Staff engagement
  can be crucial for succeeding in the implementation of any patient-flow procedure.

The above variables probably do not differ from the critical success approaches necessary for supporting other kinds of improvements in quality. Hence, whatever has been learned about the implementation and endurance of the changes can be correspondingly applied to the organizations investigating the implementation of the approaches for analyzing or improving patient flow. Therefore, focus on such critical principles supports improvements better than emulation of particular process modifications at other institutions; however, examining the ways for analyzing and improving the patient flow would be an essential potential (60).

Therefore, the chosen procedure must continually be matched to the imagined manner of activity in the hospital. The present research specified gaps in the current publications and showed that NHS Trust is observed in some hospital departments. However, later investigations may expand the above list because other hospital departments may transition to the combined planning. Based on the decision stage, a few studies emphasized the strategic or tactical decision stage. Moreover, well-known approaches to resolve the NHS Trust have been considered meta-heuristics and multiagent, but the accurate approaches have lower popularity because of the problem's complexity. In addition, there are not any articles incorporating the preferences of nurses or patients.

On the one hand, our study did not deal with such topics primarily caused by the problem complexity. Furthermore, incorporating the nurses and patients' preferences could result in the combined hospital planning and elevate patient satisfaction. It has been found that some publications addressed practical implementation of the approaches, for which experts in the field can obtain few documents. For the additional expansion of the combined planning in hospitals, the obtained practical outputs should be considered for further studies.

## Data Availability Statement

The original contributions presented in the study are included in the article/supplementary material, further inquiries can be directed to the corresponding author.

## Ethics Statement

Written informed consent was not obtained from the individual(s) for the publication of any potentially identifiable images or data included in this article.

## Author Contributions

All authors listed have made a substantial, direct and intellectual contribution to the work, and approved it for publication.

## Conflict of Interest

The authors declare that the research was conducted in the absence of any commercial or financial relationships that could be construed as a potential conflict of interest. The reviewer XW declared a shared affiliation, with no collaboration, with the authors to the handling editor at the time of the review.

## Publisher's Note

All claims expressed in this article are solely those of the authors and do not necessarily represent those of their affiliated organizations, or those of the publisher, the editors and the reviewers. Any product that may be evaluated in this article, or claim that may be made by its manufacturer, is not guaranteed or endorsed by the publisher.

## Figures and Tables

**Table:** 

*a*_*ij*_	The fixed concept of the processing duration of mission *(i)* in the unit *(j);*
β_*ij*_	The varied concept of the processing times of the mission *(i)* in the unit *(j, which* expressed the time needed via the unit for processing 1 unit of the materials doing the task *(i);*
$\rho_{si}^{p},\rho_{si}^{c}$	The proportion of state *(s)* generated, which has been used by the mission *(i)*,
*B*(*i, j, n*)	The continuous amounts of the material performing the mission *(i)* in the unit *(j)* at the event point *(n)*,
*dem*_*s*_	The market demand for the state *(s)* at the end of the time horizon,
*H*	The time horizon,
*i* ∈ *I*	Missions,
*I*_*j*_	The mission that could be done in the unit (*j*),
*I*_*s*_	The mission that produced or consumed state *(s)*,
*j* ∈ *J*	Units,
*J*_*i*_	The units appropriate to conduct the mission *(i)*,
*n* ∈ *N*	The event points, which represented a task starting point;
*p*_*s*_	The price of reported (s),
*Profit*	The continuous overall profit,
*s* ∈ *S*	State,
*S*_*p*_	The states that corresponded to the resulting products,
*S*_*r*_	The states that corresponded to the raw materials,
*ST*(*s, n*)	The continuous value of state *(s)* at the event point *(n)*,
$ST_{s}^{\max}$	The available Max storage for reported *(s)*,
*STF*(*s*)	The continuous final value of reported *(s)* at the end of time horizon,
*STI*(*s*)	The continuous initial value of reported *(s)* at the starting of the time horizon,
*T*^*s*^(*i, j, n*)	The continuous time, at the mission *(i)* reported in the unit *(j)* at the event point *(n)*,
*T*^*f*^(*i, j, n*)	The continuous time, at the mission *(i)* finished in the unit *(j)* whereas it started at the event point *(n)*,
$V_{ij}^{\min}$	The Min value of the material processed via the mission *(i)* needed for starting the operating unit *(j)*,
$V_{ij}^{\max}$	The Max capacity of the unit *(j)* while processing the mission *(i);*
*wv*(*i, n*)	Binary, whether the mission *(i)* started at the event point *(n)*.

## Footnotes

^1^One of the effective ways to cut overrun costs and add to the budget is to do outsourcing. But what is outsourcing, and how it works? In outsourcing, we try to add budget and cut over cost testing through secondary prevention (trying to detect a disease early and prevent it from getting worse. Secondary prevention, comprising diagnostic tests, “e.g., mammography, ultrasonography, magnetic resonance imaging, breast self-examination, as well as modern and more precise imaging methods,” help the early detection of tumors or lesions predisposing to tumors). In an example of outsourcing, consider a hospital that has every specialist under the sun. But other hospitals can't even afford to keep one specialist on staff full time. The country is full of people with specific medical needs that their local hospital can't meet, but we can, so instead of outsourcing our patients, we're going to outsource our doctors.

^2^On-Line Analytical Processing.

^3^**Claim efficiency:** (1) Switch the nurses' scheduling to a sensitivity based-system (it rewards loyalty); (2) ED start supply chain management tracking software (for instant restocking of critical items); (3) No more double and triple shifts (personnel needs rest or people die); (4) D or D algorithm which means you have ten minutes per patient to diagnose or discharge (this keeps the patients moving through).

^4^When people are still coming in but there is no way to put them out, we go to the red alert. In this condition, the number of coming in patents is above the number going out, and there is nowhere to put them, and we haven't got any spare capacity (bed), we get to the point where we are genuinely thinking can we treat these sick people? A red alert demonstrates there is a significant risk to the patients' safety. In this condition, all non-emergency surgeries should be canceled, and we need to search for a free bed to start to discharge the beds until the flow moves forward. Whenever the hospital is on alert, all wards are under pressure to discharge the patients. What we require to do is follow the patients from inter to leave. When all wards are full, and there is no bed in any wards; at the moment, the emergency admission system is running at its absolute limit; it is starched. The point where if one person, one key person doesn't turn on for whatever the reason, then whole things just can fall apart. Just like a real domino, just one person comes out and the whole thing just collapses. Because the flow stops, you get blockage and back right away up to the front door. Everything just becomes impossible. Whenever the hospital is on alert, all of the wards are under pressure to discharge the patients.

^5^Markov decision processes.

^6^***Notice*.** in this Research for Analysis we Consider Hospital as a Supplier in (SCM) Which Is Supplying Services for Patients.

^7^We need to have a system that combines the observations for a complete answer for the observation algorithm. We refer to a bunch of blind people touching an elephant when asked, will all say it's something different. The person at the trunk will say it's a snake, the person at the leg will say it's a tree, who placed his hand upon its side said the elephant, “*is a wall*.” Another who felt its tail described it as a rope. Only by combining all of these observations do we see it's an elephant. The point is this while each blind person is proclaiming what they believe to be is an absolute truth, in fact, all of their truths are just relative based on their experience of the elephant. No one has the Truth in its entirety.

## Appendix A

### Continuous-Time Procedure Scheduling Formulation

Floudas et al. introduced the continuous time process scheduling formulation (69–71).

## Nomenclature:

***Constraints***

Allocation constraints

(A1) $$\begin{array}{l} \underset{i\in I_{j}}{\sum }wv\left(i,n\right)\leq 1,\forall_{j}\in J,n\in N \end{array}$$

(A2) $$\begin{array}{l} V_{ij}^{\min}wv\left(i,n\right)\leq B\left(i,j,n\right)\leq V_{ij}^{\max}wv\left(in\right),\forall_{i}\in I,j\in J_{i},n\in N \end{array}$$

Storage constraints

(A3) $$\begin{array}{l} ST\left(s,n\right)\leq ST_{s}^{\max},\forall_{s}\in S,n\in N \end{array}$$

Material balances

(A4) $$\begin{array}{l} ST\left(s,n\right)=STI\left(s\right)+\sum_{\in I_{s}}\rho_{si}^{c}\sum_{j\in J_{i}}B\left(i,j,n\right),\forall_{s}\in S,n\in N,n=1 \end{array}$$

(A5) $$\begin{array}{l} ST\left(s,n\right)=ST\left(s,n-1\right)+\sum_{i\in I_{s}}\rho_{si}^{c}\sum_{j\in J_{i}}B\left(i,j,n-1\right)+\sum_{i\in I_{s}}\rho_{si}^{c}\sum_{j\in J_{i}}B\left(i,j,n\right),\forall_{s}\in S,n\in N \end{array}$$

(A6) $$\begin{array}{l} ST\left(s\right)=ST\left(s,n\right)+\sum_{i\in I_{s}}\rho_{si}^{c}\sum_{j\in J_{i}}B\left(i,j,n\right),\forall_{s}\in S,n\in N,n=N \end{array}$$

Demand constraints

(A7) $$\begin{array}{l} STF\left(s\right)\geq dem_{s},\forall_{s}\in S \end{array}$$

Duration constraints

(A8) $$\begin{array}{l} T^{f}\left(i,j,n\right)=T^{s}\left(i,j,n\right)+a_{ij}wv\left(i,n\right)+\beta_{ij}B\left(i,j,n\right),\forall_{i}\in I,j\in J_{i},n\in N \end{array}$$

Sequence constraints: same mission in the same unit

(A9) $$\begin{array}{l} T^{s}\left(i,j,n+1\right)\geq T^{f}\left(i,j,n\right),\forall_{i}\in I,j\in J_{i},n\in N,n\neq N \end{array}$$

Sequence constraints: various mission in a similar unit

(A10) $$\begin{array}{l} T^{s}\left(i,j,n+1\right)\geq T^{f}\left(i^{\prime },j,n\right)-H\left[1-wv\left(i^{\prime },n\right)\right],\forall_{j}\in J,i,i^{\prime }\in I_{j},i,i^{\prime }\in I_{j},i\neq i^{\prime },n\in N,n\neq N \end{array}$$

Sequence constraints: various mission in diverse units

(A11) $$\begin{array}{l} T^{s}\left(i,j,n+1\right)\geq T^{f}\left(i^{\prime },j^{\prime },n\right)-H\left[1-wv\left(i^{\prime },n\right)\right],\forall_{j,j^{\prime }}\in J,i,i^{\prime }\in I_{j^{\prime }},i\neq i^{\prime },n\in N,n\neq N \end{array}$$

Time horizon constraints

(A12) $$\begin{array}{l} \begin{array}{c} T^{f}\left(i,j,n\right)\leq H,\forall_{i}\in I,j\in J_{i},n\in N\\ T^{s}\left(i,j,n\right)\leq H,\forall_{i}\in I,j\in J_{i},n\in N \end{array} \end{array}$$

***Objective function:***

(A13)
$$\begin{array}{l} Max\;Profit=\underset{s\in S_{p}}{\sum }p_{s}.STF\left(s\right)-\underset{s\in S_{r}}{\sum }p_{s}.STI(s). \end{array}$$
